# Will they or won't they? Understanding New Zealand adults' attitudes towards using digital interventions

**DOI:** 10.3389/fdgth.2023.1008564

**Published:** 2023-03-08

**Authors:** Holly Wilson, Penelope Hayward, Liesje Donkin

**Affiliations:** ^1^ Department of Psychological Medicine, Faculty of Medical and Health Sciences, The University of Auckland, Auckland, New Zealand; ^2^ Department of Psychology, Faculty of Health and Environmental Sciences, Auckland University of Technology, Auckland, New Zealand

**Keywords:** digital health, eHealth, mental health, digital intervention, attitude

## Abstract

**Background:**

Digital interventions deliver healthcare *via* the internet or smartphone application to support people's well-being and health. Yet uptake is relatively poor. Furthermore, several studies exploring attitudes towards digital interventions have found inconsistent attitudes. In addition to this, regional and cultural nuances may further influence attitudes to digital interventions.

**Objective:**

This study aimed to understand New Zealand adults' attitudes towards digital interventions and their influences.

**Results:**

A mixed-method design consisting of a cross-sectional survey and semi-structured interviews found that New Zealand adults hold varied and complex attitudes towards digital interventions. Attitudes were found to be influenced by group membership and the scenarios in which digital interventions are made available. In addition, beliefs about the benefits and concerns surrounding digital interventions, knowledge, perceived views of others, and previous experience and confidence influenced these attitudes.

**Conclusions:**

Findings indicated that digital interventions would be acceptable if offered as part of the healthcare service rather than a standalone intervention. Key modifiable factors that could positively influence attitudes were identified and could be leveraged to increase the perceived acceptability of digital interventions.

## 1. Introduction

E-health, using information and web-based technology to deliver healthcare (1, 2), has numerous benefits for individuals and healthcare systems. E-health has given people a greater choice, access to more information and increased ability to manage healthcare (1, 3) through the development of patient portals (4, 5) and virtual delivery methods (2, 6). Likewise, it has increased safety through more rapid and accurate communication between healthcare professionals (7, 8) and facilitating best care practices through electronic decisional aides and prompts (4, 9). Healthcare systems have also become more cost-effective through E-health (2, 10) by maximising access to resources (11, 12) and lower-cost treatment options (9).

One subtype of E-health is the delivery of structured health programmes *via* the internet or smartphone applications, termed digital interventions (DI). DI supports mental and physical health by helping people to engage in behaviours that may prevent the development of illness (13–16), facilitating early detection (15, 17, 18), and improving the management of chronic conditions (19–21). For physical health, DI can support people to engage in exercise and healthy eating (13, 14), to better self-manage their health or chronic illnesses (19–21), and improve medication adherence (21). For mental health, DI can support people to engage in well-being behaviours as prevention (15, 16) and help people who would not meet the threshold for secondary or tertiary services (15, 17, 18). Likewise, DI can deliver evidence-based psychological techniques that improve mental health (15, 22–24).

Despite the benefits of DI, real-world engagement could be better (25, 26). For instance, real-world uptake of DI that focuses on anxiety and depression ranges between 1%–28% (26). Low uptake means that potential beneficiaries are unlikely to seek out and use DI in their everyday life. Likewise, DI may not be considered a viable treatment option if there is low uptake and high attrition.

Attitudes are people's overall evaluation of a stimulus, which guide behaviour (27, 28). Poor attitudes to DI have been linked to less likelihood of engagement (19, 27–32), whilst favourable attitudes increase the possibility of use (19, 29–31). Positive attitudes have also been linked to greater benefits following DI use (33) than those with unfavourable attitudes. Poor or negative attitudes towards DI are thought to be one of the leading causes of poor uptake and attrition in usage (34, 35).

How people come to hold attitudes about DI is complex. Two key models related to attitude formation for technology are the Technology Acceptance Model [TAM; (36, 37)] and the Unified Theory of Technology Acceptance [UTAUT; (38, 39)]. According to these models, such attitudes are influenced by people's beliefs about technology, particularly regarding benefits and concerns, perceived ease of use, perceived effort to use the technology, the opinion of important others (social norms), and whether people have access to the required technology (36–39). Similarly, numerous studies have shown that people's experiences (40, 41), the influence of key social relationships (29, 41, 42), and specific beliefs held about DI (27, 28), such as the expected benefits (29, 42–46), perceived ease of use (41, 46), perceptions of data security (19, 47), and internet confidence (29, 48) influence attitudes to technology.

Despite attitudes influencing the likelihood of using DI, surprisingly, little is known about the attitudes people hold. Attitudes about DI for physical health have rarely been studied, with few existing studies suggesting somewhat positive attitudes (49, 50). Attitudes about DI for mental health have been more widely studied, perhaps as DI are more commonly used to support mental health. Evidence suggests that attitudes towards DI to support mental health vary (40, 51–54) and can differ by population or by the purpose of the DI. For instance, previous use leads to more favourable attitudes (52, 53), and people prefer using DI to support mild conditions rather than severe (40, 55). Health professionals have shown less favourable attitudes than the public (51, 56). Taken together, attitudes towards DI are complex and influenced by a range of factors that can influence uptake and use.

In New Zealand (NZ), little is known about people's attitudes towards DI. Previous research has shown that people in NZ have positive attitudes towards DI for weight loss (57) and that positive attitudes are linked to prior use (58, 59). However, despite not knowing if people want to use DI, the NZ government increasingly plans to integrate technology and DI into healthcare (60). To date, no studies have looked at NZ adults' attitudes to or intended use of DI. Given that international literature has demonstrated wide variation in attitudes and uptake, it is essential to explore attitudes to DI if there will be significant resource investment in this space. Therefore, this study sought to understand NZ adults’ attitudes towards DI and what shapes these attitudes.

## 2. Method

This study utilised a mixed-methods approach consisting of a cross-sectional survey and semi-structured interviews to explore NZ adults' attitudes towards DI and the factors influencing these attitudes.

### 2.1. Participants

Participants were NZ residents or citizens over 18 years who could speak and understand English at a level to consent to participate in the study. For the interview, participants also needed access to technology that would allow them to complete the interview by video-calling platform or telephone.

### 2.2. Recruitment

Participants were recruited online between October 2020 and March 2021. Recruitment methods consisted of unpaid advertising on social media through the researchers' networks and contacting organisations with groups of interest, such as caregivers and health professionals. To limit bias from recruiting people online who may be more comfortable using the internet, printed flyers and posters were distributed in areas with a high density of population of interest. Purposeful recruitment of specific populations aimed to obtain a representative sample of the NZ population with an emphasis on recruiting Māori, the indigenous people of NZ.

A power analysis was conducted using an online sample size calculator (surveymonkey.com) based on a population of 5,000,000 with a confidence level of 95% and a margin of error of 5%. This calculation gave a minimum sample size of a minimum of 385 participants. We aimed for a slightly higher sample size to ensure that a diverse range of participants was represented in our sample. A target sample size of 10–15 interviews was estimated to reach theoretical saturation, typically between 6 to 12 interviews. Recruitment ended when 400 participants completed the survey, and theoretical saturation was met at 14 interviews.

### 2.3. Study procedure

Participants could take part in the survey either online or in pen-and-paper format. The survey gathered information about demographics, health status, internet access, usage and confidence. Participants were then provided with the following definition: “digital interventions are programmes or tools delivered via the internet or smartphone that have a clear structure or program and target health or mental health outcomes. They could be things like apps, chatbots, or online treatment programmes”. They then rated their attitudes towards DI (37 items) and factors that may influence attitudes based on the literature (43 items). Health professionals were also asked to rate their attitudes towards using DI in their workplace and for personal use (13 items). Following the survey, participants could enter a draw to win a gift voucher as an acknowledgement for their time.

Participants who expressed interest in an interview were contacted to confirm the interview's purpose and process and their willingness to participate. Interviews took place in person or *via* Zoom or telephone, depending on participant preference and COVID-19 restrictions. Interviews lasted between 20 and 60 min. The interviews centred around one key question, “What are your views on digital interventions?” with further probing questions exploring participant attitudes about DI and the factors that contributed to these views – for example, “What experiences have you had with digital interventions?”. Each interviewee received a NZ$30.00 voucher.

### 2.4. Cross-sectional survey design

As there was no single scale that covered all items that may influence attitudes toward DI, items were developed from existing scales and key models including the TAM (36), the UTAUT (39), the extension of the UTAUT (41), the attitudes towards psychological online interventions questionnaire [APOI; (61)], and the e-therapy attitudes and process questionnaire [e-TAP; (62)]. Items were also drawn from previous studies that measured people's attitudes towards e-health (40, 44). Where relevant, items were kept in their original format (see Table 1 for item source).

**Table 1:** Questionnaire items – DI attitudes and potential influences, including the source of original items (where applicable) and corresponding item number for the current study.

Concept	Description of concept	Scales items drawn from/adapted from	Items in this study (see Multimedia Appendix 1 for the survey)
Behavioural intention	Participants’ intention to use DI in the future	A web-based acceptance facilitating intervention for identifying patient's acceptance, uptake and adherence to internet and mobile-based pain interventions: a randomised controlled trial. (44)	43–45
Knowledge	Participants’ knowledge of DI	Extension of the UTAUT (41)	41, 42
Ease of use	Participants’ perceptions of how easy a DI is to use	A web-based acceptance facilitating intervention for identifying patient's acceptance, uptake and adherence to internet and mobile-based pain interventions: a randomised controlled trial (44)	90, 92–94
		Items adapted from UTAUT (45)	
		- Item B21 - Item B22 - Item B24	86 87 91
		Generated by researchers	88, 89
Performance expectancy	Participants’ perceptions of the benefits of using a DI	Items adapted from the APOI scale (61)	
		- ABE1 - ABE2 - ABE4 - SEC1 - SEC2 - SEC3	108 109 110 111,112 113 114
Social influence	The opinion of important others about DI	Acceptability of internet treatment of anxiety and depression (40) UTAUT (38): item SI1 Generated by researchers	96–99 100 95
Effort expectancy	Participants’ perception of the effort required to engage with a DI, such as time and energy demands	(40)effort expectancy item APOI scale (61) item SEE4	103,104 102
Access to appropriate technologies	If participants had access to the technology required to use a DI	UTAUT (38) item FC1	101
Data security	Participants’ perception of how secure information would be	(40): concerns regarding data security	105, 106
Accessibility	Participants’ perceptions of privacy, flexibility, cost and convenience	Generated by researchers	107, 115–117
Internet accessibility	Participants’ accessibility to the internet	Generated by researchers	4
Internet anxiety and confidence	If people experience anxiety while using the internet and people's confidence using technology	(44): Internet anxiety items (40): Facilitating conditions items 1&2 Generated by researchers	29, 30 29, 30 28
Culturally acceptability (results presented elsewhere)	If DI fitted within participants’ culture	Generated by researchers	118–123
COVID-19	If participants’ experience with COVID-19 shaped attitudes	Generated by researchers	140–142

### 2.5. Ethical approval

Ethical approval was obtained from The University of Auckland Human Participants Ethics Committee, reference number UAPHEC3037.

### 2.6. Data analysis

The cross-sectional survey was analysed using SPSS version 27. Scores were calculated by averaging all items related to each relevant factor. This occurred for overall attitudes towards DI, attitudes to DI that support physical health (PH), and attitudes to DI that supported mental health (MH) and each factor that was hypothesised to influence attitudes. Each average score was measured on a 5-point Likert scale.

Data were examined for normality, and if the assumptions of normality were not met, nonparametric tests were used. Independent samples t-test, one-way ANOVA and chi-square tests were used to examine group differences in attitudes. Pearson's correlations and independent samples t-test were used to examine the effect of each variable on attitudes. Stepwise regressions were calculated with each item with a significant Pearson's correlation with that attitude (Overall, PH, MH).

Interviews were audio-recorded and transcribed verbatim by HW. Each interview transcript was then analysed using inductive thematic analysis to draw the themes from the interviews themselves (63). HW coded each interview to reflect key themes, while LD coded a selection of interviews. Coding occurred with hard copies or in Microsoft Word. The authors discussed codes themes to ensure agreement and consistent interpretation of themes. Where there was disagreement about themes, these were discussed and resolved.

## 3. Results

Four hundred and eight people participated in the survey; 17 (4.2%) participants were excluded as they did not meet the minimum completion rate of reporting at least their exposure to DI, leaving 391 participants for analysis (see Table 2 for demographics). Most participants were female (80.1%, *n* = 313) with an average age of 44.44 years (range: 18–86 years, *Mdn* = 43.00, *SD *= 16.99). Most participants (68.5%) identified as NZ European/Pākehā, with 13.6% identifying as Māori, 1.0% as Samoan, 3.3% as Chinese, 2.3% as Indian and 11.3% as another ethnicity. Most participants (60%) held at least a university qualification, and 99.2% reported having access to the internet at home (*n* = 388). There were no significant differences between the participants who completed the survey on paper (*n* = 35, 8.9%) or online (*n* = 356, 91.9%) in terms of demographics, self-reported health, internet access or confidence using technology.

**Table 2:** Demographics of participants excluded from analysis and those included in the final analysis*.*

		Final Sample			
	Excluded from the final analysis (*n* = 17)	Included in the final analysis (*n* = 391)	Completed the survey (*n *= 351)	Did not complete the survey (*n *= 40	Differences between those who completed and those who did not complete
Demographic	*n* (%)	*n* (%)	*n* (%)	*n* (%)	
Residency Status					x^2^(1) = 1.12, *p *= .291
Resident	13 (76.5)	341 (87.2)	47 (13.4)	37 (92.5)	
Citizen	3 (5.9)	50 (12.8)	304 (86.6)	3 (7.5)	
Not Reported	1 (5.9)	0 (0)	0 (0)	0 (0)	
Education					x^2^(11) = 8.77, *p *= .643
No formal education	2 (11.8)	19 (4.9)	17 (4.8)	2 (5.0)	
NCEA Level 1/ School Certificate	0 (0)	23 (5.9)	20 (5.7)	3 (7.5)	
NCEA Level 2/ Six Form/University Entrance	0 (0)	19 (4.9)	18 (5.1)	1 (2.5)	
NCEA Level 3/ Bursary	3 (17.6)	23 (5.9)	21 (6.0)	2 (5.0)	
Level 4 Certificate	0 (0)	19 (4.9)	19 (5.4)	0 (0)	
Level 5 Diploma	0 (0)	12 (3.1)	11 (3.1)	1 (2.5)	
Level 6 Diploma	0 (0)	11 (2.8)	11 (3.1)	0 (0)	
Bachelors	2 (11.8)	128 (32.7)	117 (33.3)	11 (27.5)	
Masters	2 (11.8)	66 (16.9)	56 (16.0)	10 (25.0)	
Doctorate	1 (5.9)	40 (10.4)	33 (33.3)	7 (17.5)	
Other	0 (0)	30 (7.7)	27 (7.7)	3 (7.5)	
Not reported	7 (41.2)	0 (0)	1 (0.3)	0 (0)	
Employment					x^2^(6) = 5.00, *p *= .543
Employed full-time	6 (35.3)	184 (47.1)	169 (48.1)	15 (37.5)	
Employed part-time	1 (5.9)	80 (20.5)	70 (19.9)	10 (25.0)	
Student	1 (5.9)	36 (9.2)	20 (5.7)	2 (5.0)	
Unemployed	0 (0)	22 (5.6)	13 (3.7)	7 (17.5)	
Sickness or disability benefit	0 (0)	13 (3.3)	29 (8.3)	0 (0)	
Retired	1 (5.9)	47 (12.0)	42 (12.0)	5 (12.5)	
Prefer not to say	1 (5.9)	8 (2.0)	7 (2.0)	1 (2.5)	
Not reported	7 (41.2)	0 (0)	0 (0)	0 (0)	
Gender					x^2^(6) = 2.066, *p *= .914
Male	2 (11.8)	68 (17.4)	63 (17.9)	5 (12.5)	
Female	8 (47.1)	313 (80.1)	278 (79.2)	27 (87.5)	
Gender Neutral	0 (0)	2(0.5)	2 (0.6)	0 (0)	
Non-Binary	0 (0)	3 (0.8)	3 (0.9)	0 (0)	
Other	0 (0)	3 (0.8)	3 (0.9)	0 (0)	
Prefer not to say	0 (0)	1 (0.3)	1 (0.3)	0 (0)	
Not reported	7 (41.2)	0 (0)	0 (0)	0 (0)	
Ethnicity					x^2^(1) = .4.22, *p *= .518
Māori	0 (0)	53 (13.6)	50 (14.2)	3 (7.5)	
NZ European/Pākehā	7 (41.2)	268 (68.5)	241 (68.7)	27 (67.5)	
Samoan	0 (0)	4 (1.0)	3 (0.9)	1 (2.5)	
Chinese	0 (0)	13 (3.3)	12 (3.4)	1 (2.5)	
Indian	1 (5.9)	9 (2.3)	7 (2.0)	2 (5.0)	
Other	2 (11.8)	44 (11.3)	38 (10.8)	6 (15.0)	
Not reported	7 (41.2)	0 (0)	0 (0)	0 (0)	
Hold a community services card					x^2^(1) = 3.69, *p *= .06)
Yes	0 (0)	60 (15.4)	58 (16.6)	2 (5.0)	
No	10 (58.8)	330 (84.6)	292 (83.4)	38 (95.0)	
Not reported	7 (41.2)	0 (0)	0 (0)	0 (0)	
Caregiver					x^2^(1) = 2.11, *p *= .146
Yes	0 (0)	60 (15.3)	57 (16.2)	3 (7.5)	
No	10 (58.8)	331 (84.7)	294 (83.8)	37 (92.5)	
Not reported	7 (41.2)	0 (0)	0 (0)	0 (0)	
Currently have a mental illness					x^2^(1) = 3.73, *p *= .053
Yes	0 (0)	131 (33.7)	123 (35.0)	32 (80.0)	
No	10 (58.8)	258 (66.3)	226 (64.4)	8 (20.0)	
Not reported	7 (41.2)	0 (0)	0 (0)	0 (0)	
Currently have a physical illness					x2(1) = 2.93, *p* = .087
Yes	3 (17.6)	114 (29.2)	107 (30.5)	33 (82.5)	
No	7 (41.2)	277 (70.8)	244 (69.5)	7 (17.5)	
Not reported	7 (41.2)	0 (0)	0 (0)	0 (0)	
Health Professional					x2(1) = 1.68, *p* = .146
Yes	0 (0)	104 (29.3)	104 (29.9)	0 (0)	
No	0 (0)	251 (70.7)	247 (70.4)	4 (100)	
Internet at home					x2(1) = 1.76, *p* = .185
Yes	9 (52.94)	388 (99.2)	349 (99.4)	39 (97.5)	
No	0 (0)	3 (0.8)	2 (0.6)	1 (2.5)	
Not reported	8 (47.1)	0 (0)	0 (0)	0 (0)	
Amount of internet at home					x2(4) = 1.33, *p* = .857
Unlimited	8 (47.0)	354 (90.8)	316 (90.0)	39 (97.5)	
Capped 50–70	1 (5.9)	22 (5.6)	21 (6.0)	1 (2.5)	
Capped 25–50	0 (0)	10 (2.6)	9 (2.6)	1 (2.5)	
Capped <25	0 (0)	2 (0.5)	2 (0.6)	0 (0)	
None	0 (0)	2 (0.5)	2 (0.6)	0 (0)	
Not reported	8 (47.1)	0 (0)	0 (0)	0 (0)	
Internet access on a phone					x2(2) = 5.96, *p* = .06
Yes	9 (52.94)	375 (96.2)	337 (96.3)	38 (95.0)	
No	0 (0)	10 (2.6)	10 (2.0)	2 (5.0)	
Unsure	0 (0)	5 (1.3)	3 (0.9)	0 (0)	
Not reported	8 (47.1)	0 (0)	0 (0)	0 (0)	

No significant differences in age were observed between those who completed (*M *= 44.55, *Mdn *= 43.00, *SD* = 17.05, range = 18–86) and those who did not complete the survey [*M *= 43.50, *Mdn *= 41.00, *SD* = 16.68, range = 18–83; *t*(388) = −.37, *p* = .711].

### 3.1. Attitudes about DI

**Figure 1:**
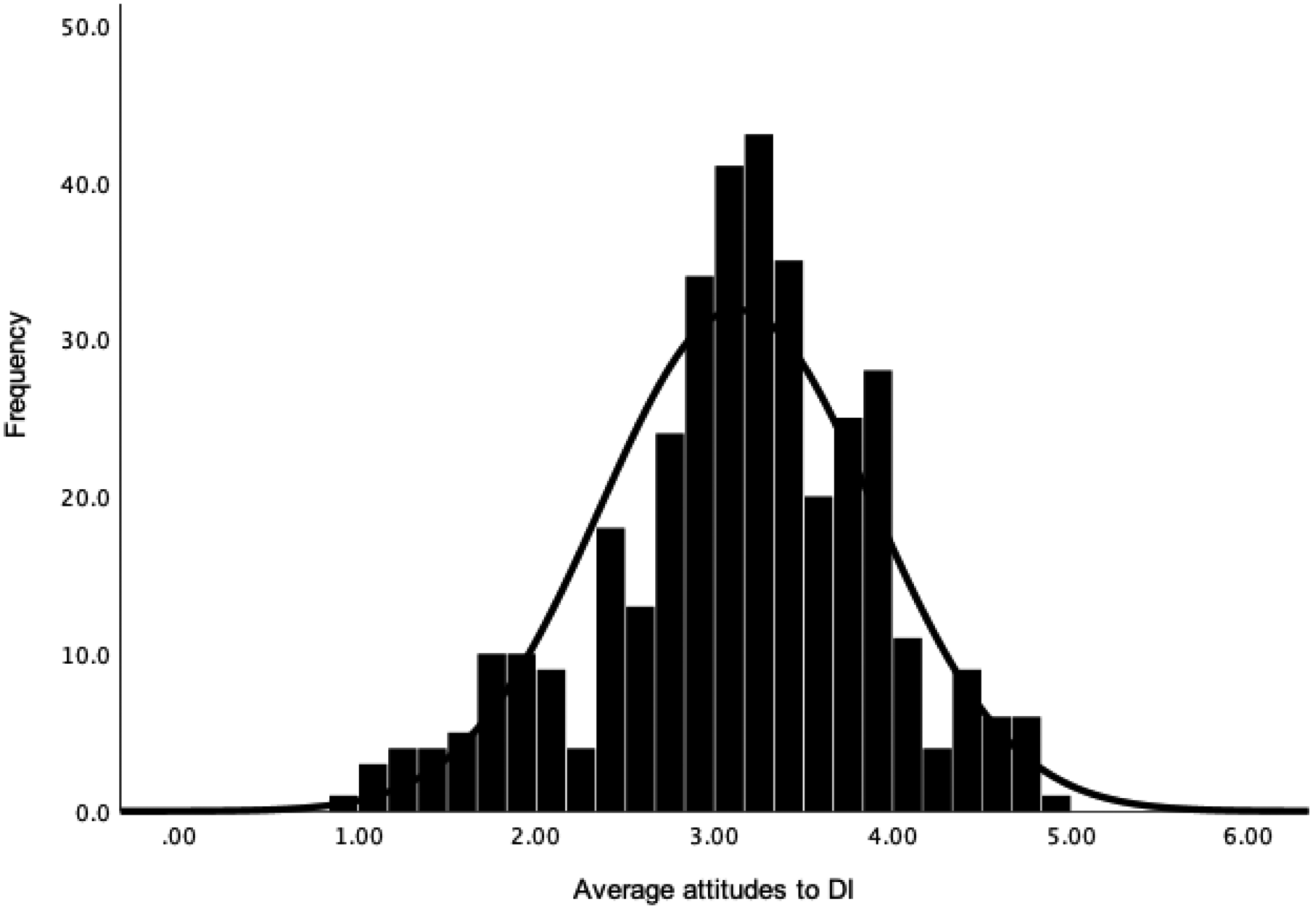
Frequency distribution with a normal curve of the average overall attitude towards DI.

**Figure 2:**
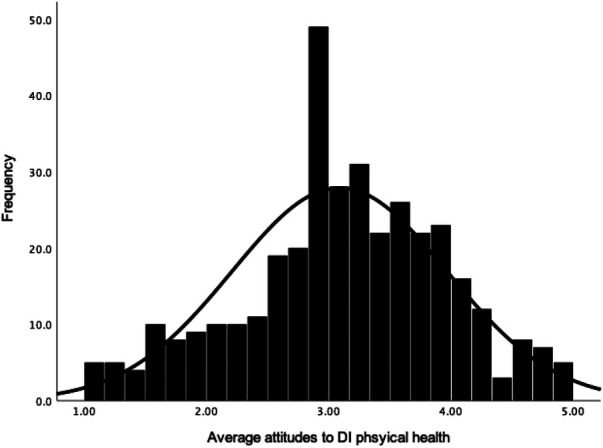
Frequency distribution with a normal curve of average attitude towards DI for physical health (PH).

**Figure 3:**
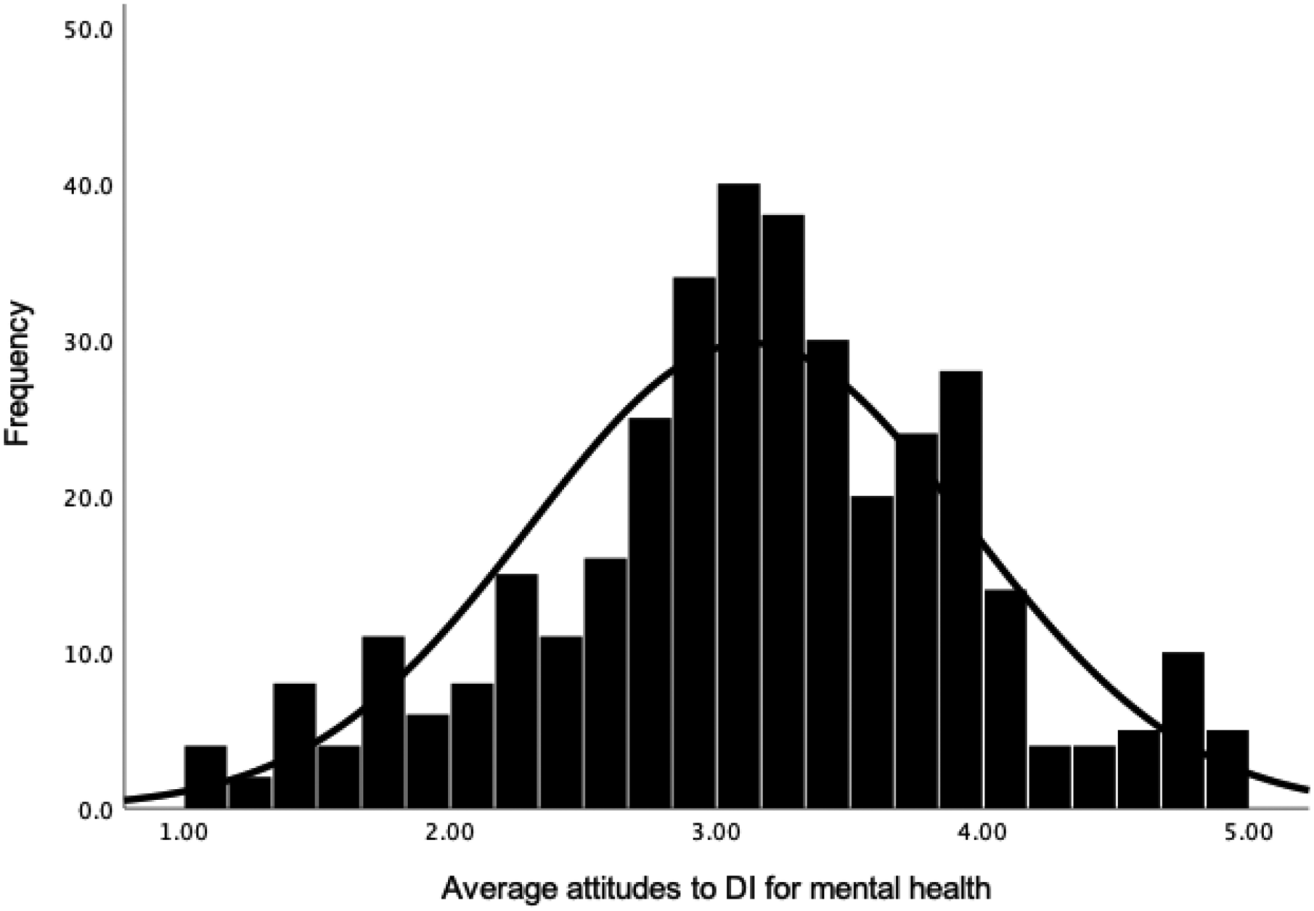
Frequency distribution with a normal curve of average attitude towards DI for mental health (MH).

Participants had an overall neutral attitude towards DI (*M *= 3.13, *Mdn *= 3.18, *SD *= 0.77, *N *= 368, see Figure 1), attitudes to DI for physical health (*M *= 3.13, *Mdn *= 3.14, *SD *= 0.82, *N *= 369; see Figure 2) and attitudes to DI for mental health (*M = *3.09, *Mdn *= 3.10, *SD *= 0.88, *N *= 369; see Figure 3). There was no significant difference between attitudes to DI for physical and mental health [*t*(368) = 1.28, *p *= .203, *d *= 0.05]. Participants' overall attitude to DI (*r* = .65, *p *< .001), attitude to DI for physical health (*r* = .59, *p *< .001) and for mental health (*r* = .61, *p *< .001) had strong positive correlations with behavioural intention to use DI in the future (*M *= 3.96, *Mdn* = 4.00, *SD* = 0.95, *n *= 369).

Participant attitudes varied by group membership (see Table 3). Health professionals had favourable attitudes toward the use of DI in their professional practice (*M* = 3.46, *Mdn *= 3.66, *SD *= 0.90, *n *= 104) and for their personal use [*M *= 3.24, *SD* = 0.82, *n *= 103; *t*(102) = −5.55, *p *< .001]. Health professionals also had more favourable attitudes to DI for mental health for their personal use (*M_MH_ *= 3.24, *SD *= 0.93; *n *= 104) than the attitudes held by the general population [*M_MH_ *= 3.02, *SD *= 0.85, *n *= 251; *t_MH_*(353) = 2.13, *p *< .05, *d* = 0.25]. No differences were observed in overall attitude *[t*(352) = 1.92, *p *= .056, *d* = 0.22] and attitudes to DI for physical health conditions [*t*(353) = 1.62, *p *= 1.07, *d* = 0.18].

**Table 3:** Differences in average attitude towards DI for group membership

Variable	Overall attitude to DI				Significant difference
	M	SD	M	SD	
Gender ^a^	Male (*n* = 67)	Female (*n *= 292)	*t*(356) = −1.49, *p *= .14, *d* = 1.09
	2.99	0.85		3.15	0.75
Community services card holder	Yes (*n* = 59)	No (*n *= 309)	*t*(72.26) = −2.83, *p *< .05, *d* = 0.45
	2.84	0.86		3.18	0.74
Distance to nearest medical centre	30 minutes or above (*n *= 11)	30 minutes or less (*n* = 358)	*t*(366) = −1.42, *p* = .160, *d* = 0.44
	3.45	0.79		3.11	0.77
Previously used a DI	Yes (*n *= 184)	No (*n *= 168)	*t*(366) = −3.62, *p *= <.001, *d* = 0.38
	3.28	0.73		2.99	0.79
Completion method	Paper (*n* = 34)	Online (*n *= 335)	*t*(35.52) = 0.86, *p *= .395, *d* = 0.27
	2.98	1.00		3.14	0.74
Informal caregivers	Yes (*n* = 58)	No (*n* = 311)	*t*(366) = 0.49, *p *= .622 *d *= 0.08
	3.17	0.89		3.11	0.74
Have a physical health condition	Yes (*n *= 111)	No (*n *= 257)	*t*(366) = 1.10, *p *= .273, *d* = 0.13
	3.06	0.79		3.16	0.76
Have a mental health condition	Yes (*n* = 127)	No (*n* = 240)	*t*(364) = −0.80, *p *= .423, *d* = 0.09
	3.17	0.81		3.10	0.75

^a^Other genders were not included in the analysis as the sample size was too small.

Participants who lived rurally had slightly more favourable attitudes towards DI than those living in urban centres, but this was insignificant (*p *= .106). Participants who held a community services card (those with low income or are high users of health services) had a significantly less favourable overall attitude to DI and for DI to support mental health (*M*_attitude_* *= 2.84, *SD *= 0.86, *M*_PH_* *= 2.91, *SD* = 0.97, *M*_MH_* *= 2.73, *SD *= 1.02, *n* = 59) than participants without a community services card (*M*_attitude_* *= 3.18, *SD *= 0.74, *M*_PH_* *= 3.17, *SD* = 0.79, *M*_MH_* *= 3.16, *SD *= 0.84, *n *= 309; *t_attitude_*(72.26) = −2.83, *p *< .05, *d* = 0.45; *t_MH_*(73.40) = −3.04, *p *< .05, *d* = 0.49). There were no differences in attitudes about DI for physical health [*t*(73.43) = −1.95, *p *= .06, *d* = 0.31] between these groups. Previous use led to a more favourable overall attitude (*p *< .001).

No differences in attitudes were observed between people who completed the survey in paper format and those that completed it online (*p *= .395), those with mental (*p *= .423) or physical health conditions (*p *= .273) and those who did not, caregivers and non-caregivers (*p *= .622), between the genders (*p *= .14) or education levels (*F = *0.66, *p* = .619, est w^2^ = 0.00).

As indicated by the average rating of items, participants preferred DI to support conditions of “mild” severity for both physical and mental health than conditions that were “moderate” or “severe” (see Table 4). DI were not perceived as acceptable to support people who were suicidal (*M* = 2.33, *SD* = 1.28, *n *= 370) and were a less acceptable option than medication (*M* = 2.91, *SD* = 1.18, *n *= 369) or psychological therapy (*M* = 2.52, *SD* = 1.09, *n *= 370).

**Table 4:** Differences in attitudes to DI for mild, moderate and severe mental and physical health conditions.

	Mild		Moderate		Severe		
	*M*	*SD*	*M*	*SD*	*M*	*SD*	F test
Physical Health (*n* = 370)	2.98	1.16	2.30	1.01	1.57	1.03	*F*(1.58,58.64) = 270.66, *p* < .001
Mental Health (*n* = 369)	2.90	1.2	2.22	1.06	1.52	1.04	*F*(1.47,539.55) = 293.00, *p* < .001

### 3.2. Factors that were associated with people's attitudes

Several factors were associated with attitudes (see Table 5). Age had weak to moderate negative correlations with overall attitude to DI (<.001) and attitude to DI for physical health (<.001), but not the attitude to DI for mental health (*p *= 0.69). Knowledge about DI (*M* = 3.66, *Mdn = *4.00 *SD* = 1.24, *n *= 370) had significant weak to moderate correlations with overall attitude (*p *< .001), attitude to DI for physical health (<.001) and DI for mental health (*p *< .001).

**Table 5:** Pearsons correlations between factors shaping overall attitude, attitude towards DI for physical health (PH) and attitude towards DI for mental health (MH).

Variable	Overall attitude		Attitude to DI for physical health		Attitude to DI for mental health	
	*r*	*p*	*r*	*p*	*r*	*p*
Average age	−.19	<.001	−.10	.069	−.25	<.001
Average knowledge of DI	.33	<.001	.25	<.001	.29	<.001
Average accessibility of DI	.67	<.001	.61	<.001	.65	<.001
Average social influence	.55	<.001	.53	<.001	.52	<.001
Average how easy a DI was to use	.51	<.001	.45	<.001	.50	<.001
Average perceptions of effort to use a DI	−.39	<.001	−.37	<.001	−.35	<.001
Average performance expectancy	.53	<.001	.51	<.001	.50	<.001
Average data security	.34	<.001	.35	<.001	.34	<.001
Confidence in using the internet	.21	<.001	.19	<.001	.20	<.001
Anxiety about using the internet	−.07	.185	−.06	.242	−.09	.098
Facilitating conditions	.23	<.001	.21	<.001	.22	<.001

#### 3.2.1. Social influence

Social influence (*M *= 3.99, *Mdn *= 4.00, *SD* = 0.67, *n *= 355) had the strongest correlation with overall attitude to DI (*p *< .001), attitude to DI physical health (*p *< .001) and DI for mental health (*p *< .001). Participants had significantly greater intention of using a DI when recommended by a doctor [*M = *4.03, *SD = *0.99*, t_d_*_octor_(369) = 4.81, *p* < .001, *d *= 0.07] or therapist [*M = *4.01, *SD = *1.03*, t_therapist_*(368) = 4.41, *p* < .001, *d *= 0.05] rather than self-seeking for something they are struggling with (*M *= 3.96, *SD = *0.95). Those who had been recommended a DI by someone close had significantly more favourable attitudes towards DI (*M* = 3.37, *SD *= 0.72, *n* = 65) than those who had not [*M* = 3.01, *SD *= 0.77, *n* = 305; *t_attitude_*(366) = 2.86, *p *< .05, *d* = 0.47].

#### 3.2.2. Perceptions about qualities of DI

Perceptions of ease of use (*M *= 3.86, *Mdn *= 3.89, *SD* = 0.88, *n = *356) had strong positive associations with overall attitude (*p *< .001), attitude to DI for physical health (*p *< .001) and DI for mental health (*p *< .001). Perception of how much effort it would take to use a DI (*M *= 2.77, *Mdn *= 2.67, *SD* = 0.85, *n *= 356) had a significant negative association with overall attitude (*p *< .001), with DI to support physical health (*p *< .001) and DI to support mental health (*p *< .001). Performance expectancy (*M *= 2.71, *Mdn* = 2.71, *SD* = 0.59, *n *= 355) had significant positive associations with overall attitude (*p *< .001), with DI to support physical health (*p *< .001) and DI to support mental health (*p *< .001). Perceptions of ease of accessibility of DI (*M *= 3.55, *Mdn *= 3.75, *SD* = 0.79, *n *= 355) had strong positive correlations with overall DI attitude (*p *< .001), attitude to DI for physical health (*p *< .001) and DI for mental health (*p *< .001).

#### 3.2.3. Technology beliefs and confidence

Low concern about data security (*M *= 3.18, *Mdn *= 3.00, *SD* = 1.01, *n *= 362) had a positive relationship with overall attitude (*p *< .001), with DI to support physical health (*p *< .001) and DI to support mental health (*p *< .001). Confidence using the internet (*M *= 4.57, *Mdn* = 5.00, *SD* = 0.67, *n *= 397) had significant positive associations with overall attitude (*p *< .001), attitude to DI for physical health (*p *< .001) and DI for mental health (*p *< .001). Anxiety about using the internet (*M *= 2.15, *Mdn* = 2.00, *SD* = 1.14, *n *= 390) had non-significant weak correlations with overall attitude (*p *= .185), attitude to DI for physical health (*p *= .242) and DI for mental health (*p *= 0.89). Access to necessary technology (*M *= 4.67, *Mdn* = 3.75, *SD* = 0.69, *n *= 355) was associated with a more favourable attitude overall (*p *< .001), with DI to support physical health (*p *< .001) and DI to support mental health (*p *< .001).

#### 3.2.4. Impact of COVID-19

Most participants reported that the COVID-19 pandemic did not change their intention to use DI (53.1%, *n *= 187); however, 42.3% (*n* = 1490) of participants reported being more likely to use DI as a result of the pandemic.

#### 3.2.5. Factors influence beliefs about DI

Participants with a community services card had a significantly lower perception of performance expectancy (*M *= 2.54, *SD* = 0.68, *n *= 58) than those without [*M =* 2.75, *SD* = 0.56, *n =* 296; *t*(352) = −2.52, *p* < .05, *d* = 0.57] and less knowledge about DI (*M *= 3.15, *SD* = 1.37, *n *= 59) than those without a community services card [*M *= 3.75, *SD* = 1.20, *n *= 310; *t*(367) = 3.42, *p* < .001, *d* = 0.35]. Health professionals had significantly greater self-reported knowledge of DI [*M*_health professionals_* *= 4.11, *SD* = 0.95, *n *= 104; *M*_public_* *= 3.48, *SD* = 1.29, *n *= 251; *t*(257.39) = 5.06, *p* < .001, *d* = 0.52]. Participants under the age of 40 had greater performance expectancy (*M *= 4.16, *SD* = 0.68, *n *= 157) than those aged between 41 to 64 years (*M *= 3.76, *SD* = 0.84, *n *= 150), while those aged over 65 had poorest performance expectancy [*M *= 3.86, *SD* = 0.85, *n *= 48; *F*(2,352) = 26.60, *p *< .001, est w^2^ = 0.13]. People who had previously used a DI placed less importance on social influence (*M *= 4.10, *SD* = 0.62, *n *= 168) and had greater knowledge (*M *= 4.18, *SD* = 0.94, *n *= 176) than participants who had not used a DI (*M*_social influence_* *= 3.89, *SD* = 0.73, *n *= 187; *t*(353) = −2.87, *p* < .05, *d* = 0.31; *M*_knowledge_* *= 3.19, *SD* = 1.03, *n *= 194; *t*(368) = −8.33, *p* < .001, *d* = 1.00).

#### 3.2.6. Predicting attitudes to DI

Stepwise regressions were used to predict attitudes toward DI. For each regression, all items that had significant correlations with the attitudes type were entered into the model. The stepwise regression (Table 6) showed that the recommendation of someone close, the approval of those close, the flexibility, and the perceived effectiveness of DI were the most important in shaping people's overall attitude [*F*(15,334) = 57.67, *p* < .001, est w^2^ = 0.71]. For attitudes to DI for PH, the approval of those close, the perceived effectiveness, the privacy and the cost were the best predictors of participants' attitudes [*F*(10,339) = 65.90, *p* < .001, est w^2^ = 0.65]. Finally, for attitudes to DI for MH, the recommendation of someone close, the approval of those close and the perceived effectiveness of the DI were key predictors of participants' attitudes [*F*(11,338) = 62.77, *p* < .001, est w^2^ = 0.66].

**Table 6:** Stepwise regression of overall attitude to DI, DI for physical health (PH) and for mental health (MH).

Variable	*B*	95%CI for *B*		*SE B*	R	R^2^
		LL	UL			
Overall attitude to DI						
					.85	.71
Item 95: I would use a digital intervention if recommended by someone close to me	0.18***	0.12	0.23	0.30		
Item 99: Those people who are important to me would approve of me using digital interventions for physical health	0.12***	0.05	0.19	0.17		
Item 115: A digital intervention is appealing because of their flexibility	0.10***	0.04	0.15	0.13		
Have you previously used a digital intervention	0.10*	0.01	0.19	0.06		
Item 112: I do not expect digital interventions to for physical health to be effective in the long term	−0.09**	−0.15	−0.04	−0.13		
Item 116: A digital intervention is appealing because of their cost	0.08**	0.03	0.13	0.10		
Item 107: A digital intervention is appealing because of privacy	0.08**	0.01	0.12	0.10		
Item 113: By using a digital intervention, I would not need professional support	0.08**	0.02	0.11	0.09		
Item 98: Those people who are important to me would approve of me using digital interventions for mental health	0.08*	0.01	0.14	0.10		
Item 96: Other people would think badly about me if I would use a digital intervention or mental health problems	0.07**	0.02	0.12	0.10		
Item 122: People from my culture use digital interventions	0.07**	0.02	0.11	0.10		
Item 111: I do not expect digital interventions for mental health to be effective in the long term	−0.07*	−0.13	−0.01	−.09		
Item 90: I would know where to get help if using a digital intervention	0.06**	0.21	0.10	0.10		
Item 102: Digital interventions could increase isolation and loneliness	−0.05*	−0.09	−0.001	−0.07		
Item 108: By using a digital intervention, I can reveal my feelings more easily than with a therapist	0.04*	0.01	0.09	0.07		
Attitudes towards DI for physical health						
					.81	.66
Item 99: Those who are important to me would approve of me using digital interventions for my physical health	0.23***	0.17	0.28	0.29		
Item 112: I do not expect digital interventions for physical health to be effective in the long term	−0.21***	−0.26	−0.15	−0.27		
Item 95: I would use a digital intervention if recommended by someone close to me	0.15***	0.09	0.21	0.18		
Item 107: A digital intervention is appealing because of their privacy	0.11***	0.04	0.15	0.12		
Item 116: A digital intervention is appealing because of their cost	0.10***	0.04	0.15	0.12		
Item 110: By using a digital intervention, I would not have to fear that someone will find out that I have a psychological or mental health problem	0.06**	0.02	0.11	0.09		
Item 113: By using a digital intervention, I would not need professional support	0.06*	0.01	0.12	0.77		
Item 90: I would know where to get help if using a digital intervention	0.06*	0.01	0.10	0.09		
Item 122: People from my culture use digital interventions	0.06*	0.01	0.11	0.08		
Item 102: Digital interventions could increase isolation and loneliness	−0.05	−0.09	−0.01	−0.07		
Average attitude towards DI to support mental health						
					.82	.67
Item 95: I would use a digital intervention if recommended by someone close to me	0.19***	0.12	0.26	0.22		
Item 111: I do not expect digital interventions for mental health to be effective in the long term	−0.18***	−0.23	−0.12	−0.22		
Item 98: Those people who are important to me would approve of me using digital interventions for mental health	0.15***	0.09	0.22	0.18		
Item 115: A digital intervention is appealing because of their flexibility	0.11***	0.04	0.18	0.13		
Item 122: People from my culture use digital interventions	0.10***	0.04	0.15	0.12		
Item 116: A digital intervention is appealing because of their cost	0.10**	0.04	0.17	0.12		
Item 90: I would know where to get help if using a digital intervention	0.09***	0.04	0.14	0.13		
Item 113: By using a digital intervention, I would not need professional support	0.08**	0.02	0.14	0.92		
Item 109: I would be more likely to tell my friends that I use a digital intervention than that I visit a therapist	0.07*	0.10	0.12	0.09		
Item 108: By using a digital intervention, I can reveal my feelings more easily than with a therapist	0.05	−0.00	−0.11	−0.08		
Age	−0.01*	−0.01	−0.01	−0.09		

CI = confidence interval; *LL = *lower limit; *UL* = upper limit, ***p < .001, **p < .01, *p < .05.

### 3.3. Qualitative findings

Twenty-one people l were interested in participating in interviews and were contacted to participate. Fourteen people took part in the interview (see Table 7 for demographics), with 71.4% (*n* = 10) being female and 71.4% (*n* = 10) identifying as NZ European/Pākehā. The average age was 43.60 years (range 27–64 years).

**Table 7:** Demographics of semi-structured interview participants

Interview Number	Age	Gender	Ethnicity
1	55	Female	NZ European/Pākehā
2	27	Female	NZ European/Pākehā
3	64	Female	NZ European/Pākehā
4	–	Female	NZ European/Pākehā
5	–	Male	Māori
6	37	Female	Pasifika/Māori
7	–	Female	NZ European/Pākehā
8	37	Female	Māori
9	–	Female	NZ European/Pākehā
10	36	Male	Māori
11	60	Male	NZ European/Pākehā
12	57	Female	NZ European/Pākehā
13	28	Male	NZ European/Pākehā
14	35	Female	NZ European/Pākehā

#### 3.3.1. People’s attitudes towards DI are varied

Eleven participants had positive attitudes towards DI. However, for two participants, having a positive attitude did not mean that they believed they would use a DI in the future, but rather that they could see the benefits and appeal of DI for others. Several participants also identified concerns about DI despite being positive about them –leaving them unsure if they would use them. Only one participant had negative views about DI and no interest in using it. Participants frequently explained that the key influence on their positive attitude towards DI was its perceived benefit.

“*I don’t particularly like [DI]”* [Interviewee #1]

“*I actually really like it, yeah, cause… umm… it actually helps me to keep track of my, like, good days and bad days.”* [Interviewee #7]

All participants had clear views about the role of DI and where DI should sit in healthcare delivery. For example, DI were perceived to need to supplement existing healthcare and not replace existing services, with many participants identifying that face-to-face healthcare was gradually moving to a DI format. Similarly, participants believed DI would be the most beneficial in supporting mild illnesses or conditions. At the same time, there were perceptions that DI would be inadequate or risky in complex or high-risk situations. Therefore, participants may be reluctant to use DI when considering their condition as “severe” or “high-risk”, and being offered a DI in this situation may invalidate their concerns.

“*…[DI] probably needs to be followed up with the health professionals, either during, after or both.”* [Interviewee #9]

“*When you are in a state of crisis, I don’t think DI would help at all. Even just the breathing apps or anything like that like… it's just that you’re not going to focus on that.”* [Interviewee #6]

“*I can see a marrying up between DI and traditional interventions.”* [Interviewee #1]

#### 3.3.2. Factors that shaped people's beliefs about DI

Attitudes about DI were shaped by participant beliefs, knowledge, and experience with DI. Specifically, people's beliefs about the benefits and risks of DI, experience and confidence with technology, and the opinions of others influenced their attitudes. Where participants had a favourable experience with DI in the past or knew of others that had good experiences, they were more likely to have favourable attitudes towards DI and to reduce anxiety about its use.

#### 3.3.3. Perceived benefits of DI above traditional healthcare shaped positive attitudes

All participants reported several benefits of DI when compared to traditional services. These benefits included increased control and choice over traditional services and the convenience and flexibility of DI rather than fixed treatment appointments and requirements. Likewise, DI provided immediate support and could be completed without disclosing their health status to their workplace or educational institution. DI were therefore perceived as offering improved anonymity, reduced travel costs, and healthcare services without waiting times. Benefits were particularly pronounced for people who lived rurally, as DI provided access to healthcare that may not be available locally. For the six participants who had used DI, the benefits they experienced while using it were crucial in shaping their attitudes.

“*It's available any time of the day and night you want to help yourself.” [Interviewee # 12]*

*“So, it does make it easier… I mean, obviously, access to these health professionals is not quite so easy when you live hours away from them … but I’m getting the best level of input I can get without having to travel hours to get to them so it's creating greater access to the assistance I need.” [Interviewee # 14]*

*“I think if someone was worried about going to see another person and shy it could help, because they’re very anonymous.” [Interviewee # 13]*

#### 3.3.4. Concerns about problem avoidance and exacerbation of health concerns influence negative attitudes

However, despite most participants having favourable attitudes towards DI and perceiving many benefits of DI, twelve participants still had concerns. Concerns commonly expressed were that DI could enable people to avoid health concerns or problems more easily than they could in traditional healthcare. Specifically, participants believed that it was easier to be avoidant with an object or a tool than with a health professional. Likewise, participants expressed concerns that a lack of engagement with DI could be detrimental and that there was no safety net around this – such as no accountability or individual follow-up. Additionally, DI were perceived to have the potential to enable self-diagnosis of symptoms or reinforce harmful beliefs that could fuel anxiety and distress.

“*It's not face-to-face, so you’re not sitting in someone's office, they might, a therapist, for example, may not pick up on the emotion of the person, that the person is going through, how it's impacting them, their body language, those certain things, that because it's not face-to-face you don’t see what you will miss out on and it could interfere with diagnosis or something… yeah… they could be underdiagnosed, it would be my only fear.” [Interviewee #12]*

*“You have to be careful that you don’t say you have something wrong with you and then you make yourself have something wrong with you.” [Interviewee #11]*

#### 3.3.5. Security and privacy concerns shaped negative attitudes about DI

Another common concern of DI was the security and management of information within the DI itself. Eight, generally older participants, expressed concern that their data would not stay private and could be shared with organisations or people with malicious intentions. Those who indicated this often had concerns about wider internet use and acknowledged that these anxieties were not limited to DI. Despite participants having some concerns about DI, they were still open to using them in the future and were hopeful that DI would benefit users.

“*I would like the assurance that whatever goes on the app stays there … I don’t want [welfare service] to know everything. You know like… and I don’t feel safe sharing everything because of them.” [Interviewee # 6]*

#### 3.3.6. More knowledge about DI would shape more favourable attitudes

Eight participants reported that they had low awareness of DI, and this made it difficult to discuss their attitudes. Low awareness was also identified as a barrier to engagement. However, despite a lack of knowledge, participants reported that more knowledge about DI, particularly the endorsement of experts in the field or health professionals, would shape more favourable attitudes. Knowledge about the effectiveness of DI was also identified as important as it could help people navigate to good quality DI, where many currently available apps are of poor quality and lack an evidence base.

#### 3.3.7. Positive beliefs about technology mean positive attitudes towards digital intervention

Participants' beliefs about using technology shaped their attitudes towards DI. Participants who were confident using technology (seven participants), usually with substantial technological experience, had favourable attitudes towards DI. However, people who were anxious or had poor confidence using technology (three participants), usually with less technological experience, were likelier to have unfavourable attitudes. Although this was not age-specific, older participants often held these concerns. However, it was also noted that assumptions should not be made based on age, as some older participants felt comfortable with the technology.

“*I use the internet quite frequently, so for an older person, it might be a bit different, but for me, it is kind of a go-to for finding out information.” [Interviewee #13]*

*“Well, when we were brought up, there were no phones or iPads or things like that, so as time moves on, things change, and maybe we’re too lazy to keep up with it or couldn’t be bothered just let it slip over us.” [Interviewee #11]*

Eight participants cited that DI had to be easy to use in terms of set-up, interaction with the app, accessible language and effective use of people's time. People who believed that DI were complex or complicated to use, or had used one with poor functionality or high levels of jargon in the past, were less likely to have favourable attitudes towards DI.

One critical factor in shaping seven participants' technology beliefs, seemingly due to increased exposure to technology, was the COVID-19 lockdowns. The restrictions on movement and access to healthcare meant greater exposure to and engagement with e-health, such as virtual consultations. This increased experience with e-health resulted in participants having greater confidence in using technology. Many participants indicated that this had influenced their attitudes and that, accordingly, they could see the benefits and appeal of DI when face-to-face healthcare was not possible.

“*COVID and going into lockdown and having to do meetings over zoom and this new world… it has opened up for us … and I think that has definitely made DI a little bit easier for me to understand and work out what was the best format of it to help me.” [Interviewee # 14]*

#### 3.3.8. The opinion of important other people influences attitudes about DI

Recommendations from close friends, health professionals or experts established the belief that DI were trustworthy and potentially beneficial for seven participants. Likewise, for participants who had used a DI, deciding to use one was often motivated by the recommendation of a trusted friend or health professional. Recommendations (or criticism) by healthcare professionals were compelling. Thus, social influence is a critical motivating factor for positive attitudes and the possible use of DI.

“*If it is a professional recommending [DI], you’re more likely to trust it rather than finding something online and relying on that source.” [Interviewee #13]*

## 4. Discussion

This study was the first to explore NZ adults' attitudes about DI and what shapes these attitudes. In line with international findings, we found that attitudes towards DI are complex and ambivalent. Attitudes varied by group membership, with health professionals and people in rural areas reporting more favourable attitudes towards DI. Low-income or high-health need populations had less favourable attitudes. People want DI to be used alongside traditional healthcare, not as a replacement, and to support people with mild health conditions rather than complex or high-risk situations, such as when someone is suicidal. Some people, despite favourable attitudes, would not personally use a DI, often due to other concerns or a lack of motivation to engage with technology. People's beliefs about perceived benefits, concerns, technology, previous experience with DI, knowledge and social norms were the most important influences on their attitudes. Specifically, beliefs about the benefits of accessibility, flexibility and convenience of DI over traditional healthcare were associated with more favourable attitudes. Despite this, people had concerns about the lack of accountability around engagement leading to low motivation and potentially detrimental effects. People also held concerns about the security and privacy of the information provided. Social norms were associated with favourable attitudes. Specifically, the endorsement of an expert in the field or a health professional encouraged people to use a quality DI. Likewise, people's beliefs about strong data security, confidence and low anxiety about the internet, and access to the necessary technology, were associated with more favourable attitudes.

These findings are consistent with previous research that demonstrates variation in attitudes (40, 54) by group membership (51–53, 56) and the comfort with DI within healthcare systems (40, 55, 56, 64). Conflicting with previous findings (51, 56), health professionals in NZ had more favourable attitudes towards DI for their personal use to support mental health compared to attitudes held by the general population. This may be due to greater professional exposure to DI or technology in their workplace; alternatively, DI may offer more confidentiality for healthcare professionals. People who held a community services card in NZ had slightly less favourable attitudes, which may be due to difficulties accessing DI and technology due to financial hardship. Alternately, it could be that low-income earners are also high healthcare users, so they are familiar with and prefer a traditional healthcare model. Equally, high service use may be associated with frustration with healthcare systems, such that DI might be perceived as “fobbing them off”.

The range of attitudes, and the belief that DI should supplement traditional healthcare, are consistent with previous literature (40, 49, 50, 54). Similarly, participants believed that DI is likely to benefit people with mild conditions (40, 55, 56), further suggesting that healthcare consumers want DI to complement the existing healthcare system rather than replace it. In offering DI, patients have more choices in their healthcare delivery, which could increase access to services. Perhaps the belief that DI are most suitable for mild conditions is driven by insufficient knowledge of the effectiveness of DI for severe or complex conditions (22–24) or a general lack of knowledge about what DI can offer to whom. Moreover, these findings demonstrate that attitudes do not always reflect behaviour (27, 28), as some participants reported favourable attitudes but had no intention of using DI. This could be due to participants perceiving that the interviewer may hold a positive attitude about DI, thus activating a social desirability bias. Conversely, the lack of intention could also indicate that although participants were interested in DI and thought DI could be helpful, there were too many barriers, such as difficulties engaging with technology. This suggests that positive attitudes alone are not enough, and barriers to use also need to be considered in evaluating people's likelihood of engaging with DI.

Consistent with previous literature (36, 37, 39), people's beliefs about the advantages of DI, typically regarding accessibility, anonymity and convenience, were influential in shaping people's attitudes. Interestingly, beliefs in perceived effectiveness or health improvements from using the DI were not commonly mentioned as a benefit, except for those who had previously used a DI. This suggests that patients' beliefs about effectiveness are shaped by previous experience, while those without previous experience base their attitudes on the perceived practical advantages of DI, such as accessibility. Thus, the factors that shape attitudes may shift over time and with experience. Social norms were also important in influencing people's attitudes. A health professional's recommendation helped people trust in the effectiveness of the DI and influenced more favourable attitudes.

Beliefs, previous experience and social norms were associated with attitudes, which is mostly consistent with previous literature (19, 22, 47, 52, 55) and supports the validity of the UTAUT and TAM model. However, the influences on attitudes regarding DI for physical and mental health seem different. For physical health DI, the approval of others, the effectiveness and the accessibility of DI contributed the most to positive attitudes. Perhaps the improved availability of support and ease of using DI can potentially overcome physical barriers to treatment seeking, such as physical discomfort related to travel for people with physical health issues, thus increasing the appeal. Whereas, for mental health DI, a recommendation from someone close to the individual, perceived effectiveness, flexibility, and the approval of others were the most important influences on people's attitudes. Given the stigma surrounding MH, it is unsurprising that social approval and recommendations are key in attitude formation, as is flexibility to minimise the impact on one's life. For overall attitudes, recommendation and approval from someone close, flexibility, previous experience, and effectiveness were most influential in shaping attitudes. Given the commonalities here, perceived effectiveness and social norms are important in shaping people's attitudes and engagement with DI and should be highlighted in the promotion of DI.

This study adds to this literature by demonstrating that experiences, including the recent restrictions in movement due to the COVID-19 pandemic, led to increased exposure to healthcare technology, shaping more favourable attitudes. Inconsistent with previous research (29, 48, 65), experiencing anxiety while using the internet was not associated with less favourable attitudes towards DI. Again, this may reflect increased online experience during COVID-19 and participants now recognising that digital tools may be a part of mainstream life.

This study provides insight into the complexity and interaction of factors that shape people's attitudes toward DI. This study suggests that DI are not likely to be appealing or used by everyone. Instead, health promotion should target factors influencing attitudes to increase engagement. Specifically, it is essential to provide accurate information about the benefits and effectiveness of DI and concerns around maximising data security. Additionally, the recommendation of a trusted individual, health professional, or expert may also be beneficial. These improved attitudes could influence people's uptake of DI and ensure that people are accessing good quality DI. Such practical considerations are essential considerations in the context of the NZ government's plan to utilise DI (60) and could help to improve the uptake of DI. Therefore, before implementing DI, there needs to be an effort to improve people's attitudes, including focusing on the influences established here.

This study is limited as the sample does not represent all NZ adults. Specifically, the views of a well-educated, internet-connected and confident female were over-represented in this survey. Similarly, the interviews are not representative of the views of all NZ adults. These findings represent the views of a sample of NZ European/Pākehā and a small group of Māori who are diverse, while the views of other ethnic groups were not well-represented. Likewise, all participants in this study had access to the technology required to use DI, while people who do not have as easy access may hold different views. The cross-sectional nature of this study does not allow the exploration of causal relationships, and it could be that there is a reciprocal relationship between attitudes and the factors that influence them. Future research could also investigate the causal nature of these relationships in an NZ population. It is also important to note that NZ is a country that provides universal healthcare, and although a small country, it does have isolated pockets where it is difficult to access care. Due to this, the findings may not be generalisable to all other countries. As with any qualitative research, the researcher's own bias and lens may shape the interpretation of the findings; however, to mitigate/ restrict this risk, discussion was held amongst all researchers in the interpretation of findings. Despite these limitations, the large sample size and diversity of people included in the study still provide useful information on attitudes about DI.

## 5. Conclusion

In conclusion, NZ adults hold varied attitudes about DI that are shaped by a complex interaction of beliefs, previous experience and social norms. Overall positive attitudes toward DI depend upon the context and healthcare circumstances in which DI is used. Unexpectedly, while some participants had direct concerns that would prevent them from using DI, others held positive attitudes but still no intention of using DI themselves. People's beliefs about the beneficial accessibility of DI relative to traditional healthcare and security concerns were vital in shaping people's attitudes. The influence of other people helped some people to decide that DI would be beneficial for their health needs – this social influence could be a method of targeting interventions to improve attitudes towards DI. Practically, DI are potentially a viable way to support the NZ healthcare system, but that would not be accessible to all members of the population if negative attitudes were not addressed. Therefore, future research must consider how to improve NZ adults’ attitudes towards DI to optimise their benefits for people's health, including professional recommendations, modifying people's underlying beliefs about DI or increasing people's exposures to DI or health-related technology. The complex interaction of beliefs, experiences and social norms shapes people's attitudes about DI.

## Data Availability

The raw data supporting the conclusions of this article will be made available by the authors, without undue reservation. Requests should be sent to the corresponding author.
